# Universal screening versus risk‐based protocols for antibiotic prophylaxis during childbirth to prevent early‐onset group B streptococcal disease: a systematic review and meta‐analysis

**DOI:** 10.1111/1471-0528.16085

**Published:** 2020-02-04

**Authors:** GF Hasperhoven, S Al‐Nasiry, V Bekker, E Villamor, BWW Kramer

**Affiliations:** ^1^ Faculty of Health Medicine and Life Sciences Maastricht University Maastricht the Netherlands; ^2^ Department of Gyneacology and Obstetrics Maastricht University Medical Centre Maastricht the Netherlands; ^3^ Department of Paediatrics Leiden University Medical Centre Leiden the Netherlands; ^4^ Department of Paediatrics Maastricht University Medical Centre Maastricht the Netherlands

**Keywords:** Antibiotic prophylaxis, early‐onset neonatal sepsis, group B streptococcus, meta‐analysis, newborn infant, risk‐based, screening, sepsis, streptococcal infections, *Streptococcusagalactiae*, systematic review, vertical transmission

## Abstract

**Background:**

Early‐onset group B streptococcal (EOGBS) disease (including sepsis, meningitis, and pneumonia) causes significant morbidity and mortality in newborn infants worldwide. Antibiotic prophylaxis can prevent vertical streptococcal transmission, yet no uniform criteria exist to identify eligible women for prophylaxis. Some guidelines recommend universal GBS screening to pregnant women in their third trimester (screening‐based protocol), whereas others employ risk‐based protocols.

**Objectives:**

To compare the effectiveness of screening‐based versus risk‐based protocols in preventing EOGBS disease.

**Search strategy:**

Key words for the database searches included GBS, *Streptococcus agalactiae*, pregnancy, screening, culture‐based, risk‐based.

**Selection criteria:**

Studies were included if they investigated EOGBS disease incidence in newborn infants and compared screening or risk‐based protocols with each other or with controls.

**Data collection and analysis:**

Risk ratios (RR) and 95% confidence intervals (CI) were determined using Mantel‐Haenszel analyses with random effects.

**Main results:**

Seventeen eligible studies were included. In this meta‐analysis, screening was associated with a reduced risk for EOGBS disease compared either with risk‐based protocols (ten studies, RR 0.43, 95% CI 0.32–0.56) or with no policy (four studies, RR 0.31, 95% CI 0.11–0.84). Meta‐analysis could not demonstrate a significant effect of risk‐based protocols versus no policy (seven studies, RR 0.86, 95% CI 0.61–1.20). In studies reporting on the use of antibiotics, screening was not associated with higher antibiotic administration rates (31 versus 29%).

**Conclusions:**

Screening‐based protocols were associated with lower incidences of EOGBS disease compared with risk‐based protocols, while not clearly overexposing women to antibiotics. This information is of relevance for future policymaking.

**Tweetable abstract:**

Meta‐analysis: general screening is associated with lower rates of early‐onset group B strep. neonatal sepsis compared with risk‐based protocols.

## Tweetable abstract

Meta‐analysis: general screening is associated with lower rates of early‐onset group B strep. neonatal sepsis compared with risk‐based protocols.

## Introduction

Early‐onset group B streptococcal (EOGBS) disease, including sepsis, meningitis, and pneumonia, is a leading cause of infant morbidity and mortality, even with a limited incidence of 0.41 cases per 1000 live births (0.32 in Asia to 0.71 in Africa), and a corresponding estimated total of 205 000 cases annually.^1^, ^2^ Group B streptococcus (GBS) is a gram‐positive commensal micro‐organism of the human intestinal tract. Vaginal colonisation was estimated in a large meta‐analysis to occur transiently in 18% (95% confidence interval [CI] 17‐19%) of pregnant women worldwide.^3^ The bacteria are transmitted vertically at delivery or earlier by ascending from the vagina into the uterus, or in some cases by invading through the intact membranes.^4^Intravenous antibiotics for at least 4 hours during labour was introduced around 1980^5^ as intrapartum antibiotic prophylaxis (IAP), and has successfully reduced vertical transmission.^6^

Women eligible for IAP are generally identified through two strategies: universal culture‐based screening for GBS colonisation, or presence of clinical risk factors for GBS transmission.^7^, ^8^, ^9^ The Centers for Disease Control (CDC) recommend universal screening for maternal colonisation between 36 and 38 weeks of pregnancy.^9^ In contrast, guidelines in the UK, the Netherlands, and New Zealand recommend risk‐based protocols.^7^, ^10^, ^11^ These clinical indicators include prolonged rupture of membranes, bacteriuria, an earlier child with EOGBS, and maternal fever. The incidence of EOGBS disease has increased in both the Netherlands and the UK in recent years.^12^, ^13^

Missed opportunities for EOGBS prevention exist in both protocols and lead to preventable infant morbidity, while overtreatment, undesirable in the light of rising antibiotic resistance and potential effects on the microbiome, occurs, too.^14^, ^15^, ^16^, ^17^ Although technical developments such as vaccines or polymerase chain reaction quick tests are promising for EOGBS prevention, they have not been widely implemented.^18^, ^19^, ^20^

Improving efficacy of IAP through either of the targeting protocols will help reduce the incidence of EOGBS disease, and may reduce overtreatment. No international consensus on the best protocol currently exists and a future strategy is under debate.^21^ As no randomised studies on the topic have been carried out, policy‐making remains a challenge.

### Objectives

The objective of this systematic review and meta‐analysis was to determine the relative success of screening‐based and risk‐based protocols in preventing EOGBS disease in newborn infants. It is, to the best of our knowledge, the only meta‐analysis comparing available data on these two policies.

## Methods

### Protocol and registration

The PRISMA statement for reporting systematic reviews was used to conduct and report this systematic review.^22^ The protocol was made public in advance in the International Prospective Register of Systematic Reviews (PROSPERO CRD42019127633).^23^ This study had no patient involvement as the review is based completely on data from the literature. 

### Eligibility criteria

#### Types of studies

Randomised or non‐randomised studies, performed in any country, on the effect of either of the two GBS prevention policies: universal culture‐based maternal GBS screening and risk‐based protocols. Studies with concurrent as well as with historical controls were considered.

#### Types of participants

The participants were all pregnant women. Outcomes were measured in all live newborn infants. No exclusion criteria were employed.

#### Types of interventions

Screening‐based protocols versus risk‐based protocols used by clinicians to determine for each individual pregnant woman whether intrapartum prophylaxis is indicated. ‘No policy’ was defined as a situation in which no consistent protocol was used but IAP could have been administered on an individual basis. Therefore, all groups included the administration of IAP, defined as intravenous antibiotic treatment intended to commence at least 4 hours before birth. All antibiotic agents (penicillin, clindamycin, etc.) used in this way and for this purpose were accepted as IAP.

#### Types of outcome measures

The outcome measure was the incidence of EOGBS disease in newborn infants as determined by positive bacterial culture from blood or cerebrospinal fluid.^24^

### Information sources

Records were obtained through literature searches in MEDLINE (using PubMed), CINAHL, and Embase databases. Additional publications were obtained manually by searching reference lists and relevant reviews.

### Search strategy

An overview of the search terms and the syntax used in MEDLINE is presented in Tables S1 and S2. Articles in English and Dutch with publication dates until 2019 were included in the final search. Last queries were run in March 2019.

### Study selection

The study selection process was performed by a primary investigator (G.H.) and critically reviewed by a second (B.K.). Records obtained from the various databases were entered in endnote X8 (Clarivate Analytics, Boston, MA, USA; 2018)^25^ to deduplicate automatically the dataset. Remaining studies were identified by titles and abstracts and were excluded if they did not fit the eligibility criteria. Remaining records were assessed by full‐text analysis, and a final selection of relevant publications was constructed. Potential disagreements between reviewers were solved through discussion and re‐evaluation. Reviewers were not blinded.

### Data collection process

Information from the studies was extracted to a pre‐defined data extraction form. Three authors of selected articles were contacted to obtain additional data.^13^, ^26^, ^27^

### Data items

The studies were indexed in the data extraction form to obtain information on four different aspects: general information on the article, data of the study population, interventions, and information on the outcomes.

### Risk of bias in individual studies

To determine the risk of bias per study, the Cochrane Risk of Bias in Non‐randomized Studies of Interventions (ROBINS‐I) tool was used, as this is the most up‐to‐date and elaborate risk of bias tool for non‐randomised studies, evaluating also the bias in historical controls.^28^ In short, seven domains of bias divided in three different time‐points in each study were scored: 

pre‐intervention—bias due to confounding; bias in selection of participants of the study;

at intervention—bias in classification of interventions;

post‐intervention—bias due to deviations from intended interventions; due to missing data; in measurement of outcomes; in selection of the reported results.

Each domain was scored Low Risk, Moderate Risk, Serious Risk, Critical Risk or No Information. To aid the judgement on the domain of ‘bias due to confounding’, common confounding factors were identified before risk of bias assessment was performed.

### Summary measures

To determine the effects of the interventions, risk ratios (RR) were calculated from the respective incidence rates in the individual studies. Incidence rates were calculated for the cases of EOGBS disease relative to the population of live births. They were expressed as cases per 1000 live births and were extracted from the studies or calculated using the data provided in the studies. If both incidence rates and absolute numbers were given, calculations were reproduced to check for incongruences.

### Methods of analysis

Studies were combined and analysed using THE COCHRANE COLLABORATION REVIEW MANAGER ( The Nordic Cochrane Centre, The Cochrane Collaboration, Copenhagen, Denmark).^29^

Due to anticipated heterogeneity, summary statistics were calculated with a random‐effect model. The Mantel‐Haenszel risk ratios with 95% confidence intervals (CI) were calculated from the data provided in the studies. Statistical heterogeneity was assessed by the *I^2^* statistic, which describes the proportion of total variation that is due to heterogeneity beyond chance.^30^, ^31^ Heterogeneity was considered high if the *I* ^2^ was above 50%. We carried out publication bias analyses (visual inspection of the funnel plot and Egger’s regression test) for meta‐analyses including at least 10 studies.^32^ The pooled number needed to screen and weighted IAP rates were calculated *post hoc*, using Microsoft EXCEL (Microsoft, Seattle, WA, USA). 33

## Results

### Study selection

The selection process is presented visually in flow diagram Figure S1. The various database searches together provided 934 citations. Three additional titles were identified from reference lists of relevant literature sources. Titles and abstracts were reviewed for 878 records, after automatically removing 59 articles. After exclusion of articles that did not meet inclusion criteria (*n* = 845), 33 articles were reviewed in detail. Sixteen articles were then excluded for reasons shown in Table S3. A total of 17 observational studies could be included in the systematic review. Data for the meta‐analysis could be extracted from 14 studies^12^, ^34^, ^35^, ^36^, ^37^, ^38^, ^39^, ^40^, ^41^, ^42^, ^43^, ^44^, ^45^, ^46^. Additional data for the meta‐analysis were kindly provided by the authors of one study.^13^ No randomised controlled trials (RCTs) on this subject were found.

### Study characteristics

Eleven studies^27^, ^34^, ^35^, ^39^, ^40^, ^41^, ^42^, ^43^, ^45^, ^46^, ^47^ provided a direct comparison of screening‐based versus risk‐based protocols (analysis 1), of which three also studied incidences during ‘no policy’ periods.^35^, ^43^, ^45^ In analysis 2 (any policy versus no policy) the introduction of universal screening^26^, ^35^, ^38^, ^43^, ^45^ was investigated in five studies and the introduction of risk‐based protocols in seven.^12^, ^13^, ^35^, ^36^, ^37^, ^43^, ^45^ (Tables 1 and 2).

**Table 1:** Study characteristics of analysis 1 (studies comparing screening‐based protocols with risk‐based protocols, using either historical or concurrent controls) and primary outcomes: incidences of EOGBS disease under different policies

Authors	study design	Controls	Country	Setting/data source	EOGBS disease definition	Screening‐based: criteria for IAP	Risk‐based: criteria for IAP	Antibiotic agent	EOGBS disease incidence per 1000 live births [95% confidence interval]		
									No policy	Risk policy	Screening
Angstetra et al. (2007)	Retrospective cohort study	historical	Australia	One tertiary obstetric unit	Blood culture ++ and necessity for admission to N(I)CU with ABs + ventilation <7 days.	34–37 weeks. Culture = pos or risk factor: previous GBS, GBS bacteriuria, preterm labour <37 weeks, temp.>38°C, prolonged ROM >18 h (if GBS status unknown) ‐> IAP	NI	benzylpenicillin IV 1.2 g‐ 600 mg/h OR clindamycin IV 600 mg/8 h OR cephalothin IV 2 g ‐ 1 g/h	n/a	0.84 [0.57–1.2]	0
Chen et al. (2005)	Retrospective cohort study	historical	USA	One tertiary care centre	Positive blood culture <7 days	35–37 weeks, vaginal and rectal positive culture ‐>IAP	Preterm birth (threat), fever (non‐specified), prolonged rupture of membranes ‐>IAP	Ampicillin or clindamycin; later penicillin G OR erythromycin	2.0 [1.5–2.6]	1.1 [0.80–1.5]	0.36 [0.24–0.55]
Edwards et al. (2003)	Retrospective cohort study	historical	US (Florida)	One general hospital	Positive blood culture <7 days	CDC guidelines of 1996	NI	Ampicillin until 1995, then penicillin	n/a	1.7 [1.0–2.9]	1.0 [0.52 –1.9]
Eisenberg et al. (2005)	Retrospective cohort study	concurrent	US (Tennessee)	All acute care hospitals in four major counties of Tennessee	Positive blood or CSF culture <7 days	CDC guidelines (retrosp. selection: found any GBS status in record, taken at least 2 days before birth ‐> screening group)	Risk group = not screened. Preterm <37, ROM> 18 h, temp. >38ºC, GBS bacteriuria, previous GBS	NI	n/a	0.85 [0.58–1.3]	0.40 [0.21–0.74]
Gilson et al. (2000)	Retrospective cohort study	Concurrent	US (New Mexico)	One hospital	Positive culture from blood, CSF or other fluid.	Women with known GBS + status, and unknown status ‐> risk factors	Rom> 18 h, temp. >38 ºC, GBS bacteriuria, previous GBS (NB: preterm infants excluded)	Ampicilin 2 g + 2 g/6 h IV before 1995; Penicillin G IV 5 ml units + 2.5 ml units/4 h	n/a	1.49 [0.56–4.0]	0
Gopal Rao et al. (2017)*	Retrospective observational study	historical	London, UK	One general hospital	Positive blood or CSF culture <7 days	Screening offered to all women in the population. According to CDC (35–37 weeks). Not screened ‐> risk group	previous GBS child; GBS bacteriuria; temp. > 38 ºC; chorioamnionitis	Benzylpenicillin 3 g IV – 1.5 g/h OR clindamycin 900 mg IV/8 h	n/a	1.1 [0.8–1.6]	0.33 [0.11–1.0]
Ma et al. (2018)	Retrospective cohort study	historical	Hong Kong	The eight hospitals of Hong Kong	Positive blood or CSF culture <7 days	35–37 weeks GA, later or at admission. 2 separate swabs: vagina and rectum. Non‐screened ‐> risk‐based. (women with previous GBS child excluded)	A previous delivery with EOGBS; GBS bacteriuria; ROM 18 h or more; GA <37 weeks; Intrapartum fever	NI	n/a	1 (not available)	0.24 (not available)
Main & Slagle (2000)	Retrospective cohort & prosp. Observational study	historical	US (California)	A primary obstetric practice + tertiary referral centre	Positive blood or CSF culture <7 days	35–37 weeks vaginal and rectal culture (+IAP for preterm)	preterm <37, ROM> 18 h, temp. >38 ºC, GBS bacteriuria, previous GBS	Ampicilin 2 g + 1 g/4 h IV OR clindamycin 900 mg/8 h	1.1 [0.58–2.3]	1.1 [0.68–1.9]	0.071 [0.010–5.0)
Schrag et al. (2002)	Retrospective cohort study	concurrent	USA	Multiple hospitals of the Emerging Infections Program Network	Pos. blood culture, CSF or other ‘normally sterile fluid’. Days unclear.	Retrospective selection: found any GBS status in record, taken at least 2 days before birth ‐> screening group	preterm <37, rupture> 18 h, temp. >38ºC, GBS bacteriuria, previous GBS	NI	n/a	0.68 [0.59–0.77]	0.33 [0.27–0.40]
Vergani et al. (2002)	Retrospective cohort study	Historical	Italy	One tertiary care centre (university hospital)	Positive blood or CSF culture <7 days	<’97 Vaginal culture between 26–28 week GA. >’97 between 35–37 week GA	Preterm <37; Rom >12 h; temp. >37.5ºC, GBS bacteriuria; previous child GBS	Ampiccilin 2 g + 1 g/4 h IV OR Erythromycin 500 mg/6 h	0.93 [0.47–1.9]	0.78 [0.39–1.6]	0.44 [0.20–0.97]
Yücesoy et al. (2004)	Prospective, quasi‐experimental	concurrent	Turkey	One antenatal clinic (in‐ and out‐patient)	Positive blood culture <72 h	Between 35–37 weeks (note: risk factor + neg. culture ‐> no IAP)	PPROM, prolonged ROM, temp. >38ºC, preterm (but individualised, tocolytics etc.)	Ampiccilin 2 g + 1 g/4 h IV	n/a	3.3 [1.0–10]	5.0 [0.71–35]

CSF, cerebrospinal fluid; EOGBS, Early‐onset Group B streptococcal infection; IAP, intrapartum antibiotic prophylaxis; IV, intravenously; NI, no information; ROM, rupture of membranes.

* Pre‐screening and post‐screening periods are taken together (cross‐over design).

**Table 2:** Study characteristics analysis 2 (introduction of any policy versus no policy) and primary outcomes: incidences of EOGBS disease under different policies

Author	study design	Controls	country	Setting/data source	EOGBS disease definition	Screening‐based criteria for IAP I	Risk‐based criteria for IAP	Antibiotic agent	EOGBS disease incidence (per 1000 live births)		
									No policy	Risk policy	Screening
Bekker et al (2014)	Surveillance study (retrospective)	Historical	NL	Netherlands Reference Laboratory for Bacterial Meningitis (all hospitals)	Positive blood or CSF culture <7 days	n/a	Previous GBS child or bacteriuria ‐> IAP. Confirmed GBS or threat preterm ‐> consider IAP. PROM + threat preterm ‐> consider GBS test	Penicillin/amoxicillin	0.11 [0.10–0.13]	0.18 [0.16–0.20]	n/a
Darlow et al. (2016)	Prospective and retrospective surveillance study	Historical	New Zealand	New Zealand Paediatric Surveillance Unit (all hospitals)	Clinical presentation + positive blood or CSF culture + laboratory indication of sepsis (CRP or leucopenia etc.). Only cases <48 h are reported.	n/a	Previous GBS child, bacteriuria, preterm, temp. >38ºC, membrane rupture> 18 h ‐> IAP	NI	0.5 [0.38–0.65]	0.23 [0.16–0.33]	n/a
Håkansson et al. (2017)	Retrospective cohort study	Historical	Sweden	National registers (all hospitals included)	Positive blood or CSF culture <7 days.	n/a	ROM> 18 h; temp.>38ºC; preterm <37 week; GBS bacteriuria; previous infant with EOGBS	NI	0.40 [0.34 –0.48]	0.30 [0.25 –0.36]	n/a
Hung et al (2018)	Retrospective cohort study	Historical	Taiwan	National Health Insurance database (all hospitals)	<7 days, GBS disease is mentioned in medical record	CDC guidelines (35–37 weeks)	n/a	NI	1.0 (not available)	n/a	0.2 (not available)
O'Sullivan et al. (2019)	Retrospective cohort study	Historical	UK	Active surveillance (all paediatricians) and laboratory databases (all)	<7 days, positive culture from blood or CSF or joint fluid	n/a	Previous GBS child or bacteriuria ‐> IAP. Preterm <37, fever> 38, PPROM, prolonged ROM> 18 h ‐> consider IAP	Penicillin/ampicillin	0.48 [0.43–0.53]	0.57 [0.52– 0.62]	n/a
Phares et al. (2008)	Retrospective cohort study	Historical	USA	All laboratories part of the Emerging Infections Program Network	<7 days, positive culture from blood or CSF	CDC guidelines	n/a	NI	0.47 [0.44–0.51]	n/a	0.34 [0.31–0.37]

CSF, cerebrospinal fluid; EOGBS, Early‐onset Group B streptococcal disease; IAP, intrapartum antibiotic prophylaxis; IV, intravenously; NI, no information; ROM, rupture of membranes.

#### Types of methods

All studies included in this review were performed according to a non‐randomised design. Most studies used national or regional microbiological data to identify cases of EOGBS disease retrospectively. Some studies included an additional exploration of the reported cases. One employed a prospective design in which medical professionals were asked to report suspected cases to the researchers^36^ in addition to confirmed cases. Some used a surveillance approach combined with one period of closer observation of a sample.^43^

#### Participants

In all studies combined, 3798 cases of EOGBS disease were identified in a total population of 11 million live births. Most studies aimed to include all pregnant women, but one study^34^ actively excluded women with previous GBS‐affected children. One study^41^ excluded preterm births to prevent risk of confounding.

#### Interventions

All studies used IAP to prevent GBS vertical transmission. Despite discrepancies in risk factors across protocols, most guidelines regarded an earlier child affected by GBS and GBS bacteriuria as a direct indication for IAP. Screening‐based protocols generally followed the 2010 CDC guidelines, consisting of a bacterial culture of a recto‐vaginal swab taken between a gestational age of 35 and 37 weeks. When maternal GBS status was unknown at labour, a risk‐based protocol was used: in the case of preterm delivery (<37 weeks GA), rupture of membranes >18 hours or maternal fever (>38°C), IAP was offered anyway. In risk‐based protocols these factors were used to determine the indication for IAP. Different antibiotics were given across studies, although most studies used penicillin and ampicillin. Alternatively, erythromycin or clindamycin was given to women allergic to penicillin.

#### Outcomes

In 14 of the 17 studies, the incidence of EOGBS disease in newborn infants was the primary outcome. Two studies had a primary focus on bacterial resistance^35^, ^39^ but included EOGBS disease as a secondary outcome. One study analysed incidences in different age groups and populations.^38^ EOGBS disease was defined by most studies as the presence of GBS in a blood or cerebrospinal fluid culture and thus included both sepsis and meningitis. Two studies^37^, ^41^ investigated incidences of ‘clinical sepsis’, too, but those were not included as cases in our review. Secondary outcomes in the studies included mortality 12, 36, 38, 45, 46 or an audit of adherence to policy 27, 43, 45

### Risk of bias within studies

Overall risk of bias was moderate in ten studies; moderate to serious in two; serious in three, critical in one, and impossible to define in one study (Figure S2, Tables S4 and S5).

Risk of bias during selection of participants was moderate in most studies, as problems arising from retrospective selection etc. were mostly controlled for. In one study,^44^ women were included in the screening group if their screening results were available at least 2 days before delivery. When authors controlled for availability of prenatal care, preterm birth etc. results remained mostly unchanged.^40^, ^44^ Risk of confounding and risk of bias at intervention, e.g. bias due to classification of interventions, were moderate in most studies. Bias post‐intervention was low or moderate in most studies. All studies with historical controls,^12^, ^13^, ^26^, ^27^, ^34^, ^35^, ^36^, ^37^, ^38^, ^39^, ^42^, ^43^, ^45^ except one using a cross‐over design,^42^ suffered from bias due to healthcare improvement over time: ‘performance bias’. All studies had possible problems due to missing data, resulting from underreported cases. In most studies this bias implied an actual greater effect of interventions.^13^

### Results of studies

Incidence rates of EOGBS disease ranged from 0.0 to 5.0 per 1000 live births, with a weighted average of 0.33/1000. The incidences are depicted in Tables 1 and 2.

**Figure 1:**
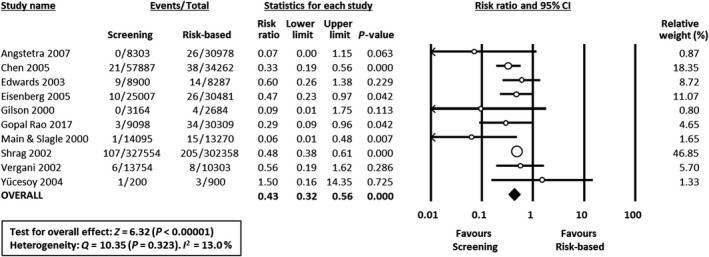
Forest plot of risk ratio (with 95% confidence intervals) of EOGBS disease (defined as positive GBS culture from a normally sterile site <7 days of age) in universal screening policy groups versus risk‐based policy groups. CI, confidence interval; M‐H, Mantel‐Haenszel.

**Figure 2:**
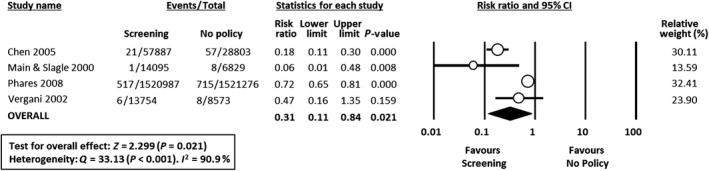
Forest plot of risk ratio (with 95% confidence intervals) of EOGBS disease (defined as positive GBS culture from a normally sterile site <7 days of age) in universal screening policy groups versus no policy groups. CI, confidence interval; M‐H, Mantel‐Haenszel.

**Figure 3:**
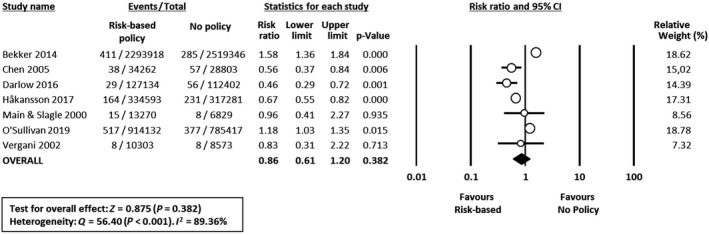
Forest plot of risk ratio (with 95% confidence intervals) of EOGBS disease (defined as positive GBS culture from a normally sterile site <7 days of age) in risk‐based policy groups versus no policy groups. CI, confidence interval; M‐H, Mantel‐Haenszel.

Risk ratios were calculated from available data in 15 studies. In two studies^26^, ^27^ the data could not be included in the meta‐analysis as absolute numbers of cases and populations could not be extracted from the record and its reference list, nor were these provided upon request. Effects of interventions expressed as risk ratios are presented in the forest plots in Figures 1, 2, 3.

The prevalence of GBS colonisation in all pregnant women in the various studies ranged between 7 and 29% (weighted mean 23%, Table S7). The pooled number needed to screen (using a pooled baseline incidence in the risk‐based group of 0.94/1000 infants) for screening versus risk‐based protocols was 1874 (Table S6). The percentage of births during which IAP was administered was reported in four studies. Weighted means were 31 and 29% in the screening and risk‐based groups, respectively (Table S7). None of the studies reported GBS resistance to penicillin or ampicillin, whereas resistance to erythromycin and clindamycin was reported by three studies (Table S7).^12^, ^35^, ^38^ Lastly, mothers of infants with EOGBS sepsis did not present any risk factors in times of risk‐based protocols in 41.3% of cases (weighted mean, Table S8). False negatives (EOBGS children born to women with negative screening results) were present in 24.2% of cases during periods of screening protocols (weighted mean, Table S8).

### Synthesis of results

Meta‐analysis showed that universal screening was associated with a reduced risk of EOGBS disease when compared either with risk‐based protocols (10 studies, RR 0.43, 95% CI 0.32–0.56; heterogeneity: *I* ^2^ = 13%) (Figure 1) or with no policy (four studies, RR 0.31 95% CI 0.11–0.84; heterogeneity: *I* ^2^ = 91%) (Figure 2). In contrast, meta‐analysis could not demonstrate a significant effect of risk‐based protocols versus no policy (seven studies, RR 0.86, 95% CI 0.61–1.20; heterogeneity: *I* ^2^ = 89%) (Figure 3). The funnel plot for analysis 1 (universal screening versus risk‐based protocols) showed slight asymmetry (Figure S3), suggesting a low risk of publication bias. Egger’s regression test did not show statistically significant asymmetry of the funnel plot (2‐tailed *P* = 0.180). Publication bias was not investigated for the other two analyses due to the low number of studies.

## Discussion

### Main findings

This systematic review summarised the available observational data on the two most common EOGBS prevention policies. Overall, our meta‐analysis shows lower incidences of EOGBS disease under screening‐based policies than under risk‐based policies. Additionally, the retrospective data show no significant EOGBS reductions resulting from the initial introduction of risk‐based policies, whereas studies on the introduction of universal screening do. Notably, two studies found higher incidences in ‘no policy’ groups compared with risk‐based policies.^12^, ^13^ The reported data on the use of antibiotics suggest similar rates of IAP under both policies.

### Strengths and limitations

The present meta‐analysis offers an up‐to‐date, international perspective on EOGBS infection prevention. Seventeen studies from ten different countries were included. By including studies in which either of two policies was introduced after a ‘no policy’ situation, their respective effects could be assessed, too.

Our study has several limitations. The limitations range from lowered blood culture sensitivity in neonatal settings per se^21^ to the different healthcare systems in which the studies were conducted. Most importantly, as meta‐analyses are dependent on the available data, the lack of randomised trials represents an important limitation. In studies with consecutive intervention groups, there was a risk of performance bias, as the observed effect could have been inherent to secular improvement of healthcare. However, reporting of cases could have improved over time, leading to a potentially even greater actual effect of screening.^13^, ^27^ In one study^42^ a period of screening was preceded and followed by a period of risk‐based management. Incidences of EOGBS disease increased to initial high levels when risk‐based policy was re‐introduced.

Another limitation lies in the variation of policies that were employed by countries and states, although the 2010 CDC guidelines were the main screening policy. Risk factors such as fever or rupture of membranes >18 hours differed and were hard to identify, both during partum and in a research setting, which is a drawback of this specific policy. Statistical heterogeneity in the direct comparison was found to be limited.

The incidence of EOGBS is generally low, and differ per country. Still, on average the numbers correlate well with data reported elsewhere,^3^ and by comparing the changes in incidences, we could nonetheless analyse the independent impacts of the two policies.

In this review, we included studies in English and Dutch from three large online databases (MEDLINE, CINAHL, and Embase). Data from potentially relevant studies in other databases or languages could have been missed, despite additional manual searching of the literature.

Of note, almost all studies controlled for possible confounders, and studies with the highest impact on the meta‐analysis^12^, ^13^, ^38^, ^44^ suffered least from risk of bias. We did not find evidence of publication bias. A last limitation of the reviewing process emerged, as risk of bias assessment was performed successively by two non‐blinded reviewers. Standardised scoring of studies, however, increased the validity of the assessment.

### Interpretation

A previously performed systematic review^48^ was limited to a smaller dataset, with the latest included study published in 2005,^40^ and did not include data on the effect of the policies compared with ‘no policy’. Our systematic review includes a meta‐analysis and presents data on eight additional studies (up to 2019), while also using the most up‐to‐date risk of bias assessment tool.^28^ Results were, however, similar. They further concur with a cost‐effectiveness Dutch study^49^ that suggested screening could protect most infants from EOGBS infection and mortality in a hypothetical cohort. The current Dutch guideline was estimated to be poorest at preventing EOGBS in that model.^49^

In this analysis, clinical risk factors were poorly associated with vertical GBS transmission. The included studies reported a significant number of missed cases (Table S8). Up to 40% of the cases of EOGBS invasive infection did not have maternal risk factors associated with them, and could therefore not be prevented under risk‐based policies. Håkansson et al.^37^ further refuted the assumption that GBS‐infected infants born to mothers without risk factors had a better prognosis, as three of 11 deaths reported in their study occurred in such circumstances.

Homer et al.^50^ concluded in a review of EOGBS disease prevention guidelines that both risk‐based and screening‐based guidelines were appropriate, but adherence to policy should be optimised to accomplish reduction of EOGBS disease. In this review, available data on adherence showed risk‐based protocols were associated with lower adherence compared with screening, which could have been a source of bias. Schrag et al.^44^ constructed a model with assumptions of perfect adherence in which screening outperformed the risk‐based protocols, suggesting the presence of an inherent advantage of screening protocols possibly owing to their limited complexity.

Importantly, concerns have been raised recently by Seedat et al.^21^ that universal screening may lead to overtreatment with the consequent increase in adverse effects and resistance to antibiotics. Our findings do not support the concern that universal screening results in antibiotic overtreatment. We found the same percentage of women receiving IAP in both protocols (Table S7). However, the evidence we found is derived from observational data in an inherently heterogeneous set of studies with possible bias. Although the estimation of possible harm by antibiotic overtreatment by Seedat et al. is relevant, the conclusions need to be verified by clinical data.^21^ We have to resign ourselves to the fact that an overtreatment may occur with both strategies. In this meta‐analysis, the overall exposure to antibiotics does not appear to differ greatly between the two protocols, whereas the rates of EOGBS disease do. Lastly, GBS susceptibility to penicillin and ampicillin seems unhindered in all studies in this review, even though some GBS strains with increasing minimum inhibitory concentrations (MICs) for beta‐lactam antibiotics have been observed elsewhere.^51^

A point of concern in both strategies is how to approach GBS prevention in preterm deliveries. It was beyond the scope of this article to compare subgroups of term and preterm infants, mostly because the available studies generally did not provide data necessary to make a subdivision. As the recommended timing for screening was between 35 and 37, and now lies between 36 to 38 weeks of gestational age (GA), infants born at 35 weeks GA or less are not covered.^9^, ^52^ Notably, these infants are at much higher risk of EOGBS disease as well as GBS‐related mortality.^53^ For this reason, the CDC has suggested that most preterm deliveries be accompanied by IAP. However, more research towards best IAP management for women delivering preterm is needed, as long‐term exposure to antibiotics poses a risk for preterm infants.^54^ Ideally, immunisation of mothers should be introduced to prevent EOGBS invasive infections, but although research in this field has been going on for years, no effective GBS vaccine has been made available so far.^18^ Until that time, it is recommended that the GBS prevention protocols are periodically re‐evaluated, and research aimed at accurate and fast detection methods for all pregnant women is continued.

## Conclusion

In this systematic review and meta‐analysis screening protocols were associated with lower rates of EOGBS disease compared with risk‐based protocols. While there is insufficient evidence to assume that risk‐based policies reduce the use of prophylactic intrapartum antibiotics, these protocols might not be able to protect infants from EOGBS disease to the same extent as general screening does. These findings can be of help to future policy‐making and individual pregnancy counselling.^55^

### Disclosure of interests

VB was a co‐author of one of the included studies in this analysis. Other authors declare no competing interests. Completed disclosure of interests forms are available to view online as supporting information.

### Contribution to authorship

GH designed and performed the search, selected studies for inclusion, collected data, planned and performed the statistical analyses, contributed to the interpretation of the results, and drafted the initial and final version of the manuscript. VB, SN and EV contributed to the interpretation of the results, and reviewed and revised the manuscript. BK conceptualised and designed the study, contributed to the search, selected studies for inclusion, supervised data collection, contributed to the statistical analyses and interpretation of the results, and reviewed and revised the manuscript.

### Funding

The authors did not receive funding or other support for this publication.

## Supporting information


**Figure S1**. PRISMA^6^ flow diagram: a visual representation of the systematic research process.

**Figure S2**. Visual representation of risk of bias assessment done using the risk of bias tool ‘ROBINS‐I’ by Cochrane. + low risk of bias; +– moderate risk; – serious risk, – – critical risk, ? no information

**Figure S3**. Funnel plot for meta‐analysis of studies comparing universal screening versus risk‐based protocols. Egger's regression test did not show statistically significant asymmetry of the funnel plot (2‐tailed *P* = 0.180).

**Table S1**. Search protocol, MeSH = Medical Subject Headings

**Table S2**. Search strategy used to identify records in MEDLINE (final search March 2019). Similar queries were run in CINAHL and Embase

**Table S3**. Articles excluded from analysis in the last step of the reviewing process

**Table S4**. Risk of bias assessment according to ROBINS‐I, in studies comparing screening‐based protocols and risk‐based protocols (analysis 1). RoB, risk of bias; PB, performance bias

**Table S5**. Risk of bias assessment: studies assessing introduction of either of the two protocols compared with a period/area of ‘no policy’ (analysis 2)

**Table S6**. Numbers needed to screen (NNS) as calculated from the data in the included studies

**Table S7**. Secondary outcomes with weighted means

**Table S8**. Missed cases in the included studies
