# Supplementary material for: Universal screening versus risk‐based protocols for antibiotic prophylaxis during childbirth to prevent early‐onset group B streptococcal disease: a systematic review and meta‐analysis
Source: BJOG. 2020 Feb 4;127(6):680–91. doi: 10.1111/1471-0528.16085 (PMC7187465; doi:10.1111/1471-0528.16085)
Supplement: Supplementary file 1 — Figure S1. PRISMA6 flow diagram: a visual representation of the systematic research process. [file BJO-127-680-s001.pdf]

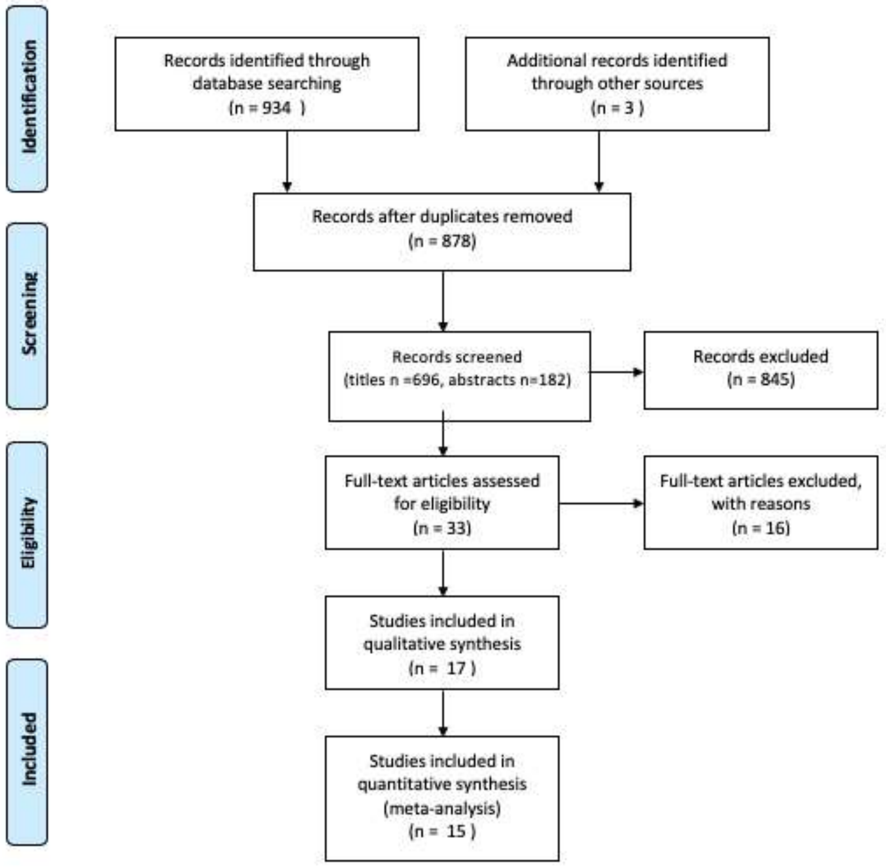

Figure S1. PRISMA[6] flow diagram: a visual representation of the systematic research process.
