# Supplementary material for: Universal screening versus risk‐based protocols for antibiotic prophylaxis during childbirth to prevent early‐onset group B streptococcal disease: a systematic review and meta‐analysis
Source: BJOG. 2020 Feb 4;127(6):680–91. doi: 10.1111/1471-0528.16085 (PMC7187465; doi:10.1111/1471-0528.16085)
Supplement: Supplementary file 2 — Figure S2. Visual representation of risk of bias assessment done using the risk of bias tool ‘ROBINS‐I’ by Cochrane. + low risk of bias; +– moderate risk; – serious risk, – – critical risk, ? no information [file BJO-127-680-s002.pdf]

	Bias due to confounding	Bias in selection	Bias in classification	Bias due to deviations	Bias due to missing data	Bias in measurement	Bias in selection of results	Overall bias
Angstetra et al.	+	+	+-	?	+-	+-	+-	+-
Chen et al.	-	+-	+-	-	+-	+	+	-
Edwards et al.	-	+-	-	-	+-	+	+-	-
Gilson et al.	+	-	+-	+	+-	+	+-	+-
Main & Slagle	+-	+	+	+	+	+	+-	+-
Schrag et al.	+-	+-	+-	+	+-	+	+-	+-
Yücesoyet al.	+-	-	+	+-	+	-	+-	-
Eisenberg et al.	-	+-	-	+-	?	+	-	+-
Vergani et al.	+-	+-	+-	+-	+-	+	-	+-
Ma et al.	-	+-	+-	+-	?	+	+-	-
Gopal Rao et al.	+	+	+-	+	+	+	+-	+-
Darlow et al.	-	+	+-	-	+-	-	+-	+-/-
Håkansson et al.	-	+	+	+-	+	+-	+-	+-/-
Bekker et al.	+-	+-	+	?	+-	+-	+-	+-
O'Sullivan et al.	+-	+-	+	+-	+-	+	+	+-
Phares et al.	+-	+-	+	?	+-	+	?	+-
Hung et al.	?	?	?	?	-	+	?	?

Figure S2. Visual representation of risk of bias assessment done using the risk of bias tool 'ROBINS-I' by Cochrane.

+ low risk of bias; +- moderate risk; - serious risk; -- critical risk; ? no information
