# Supplementary material for: Universal screening versus risk‐based protocols for antibiotic prophylaxis during childbirth to prevent early‐onset group B streptococcal disease: a systematic review and meta‐analysis
Source: BJOG. 2020 Feb 4;127(6):680–91. doi: 10.1111/1471-0528.16085 (PMC7187465; doi:10.1111/1471-0528.16085)
Supplement: Supplementary file 3 — Figure S3. Funnel plot for meta‐analysis of studies comparing universal screening versus risk‐based protocols. Egger's regression test did not show statistically significant asymmetry of the funnel plot (2‐tailed P = 0.180). [file BJO-127-680-s003.pdf]

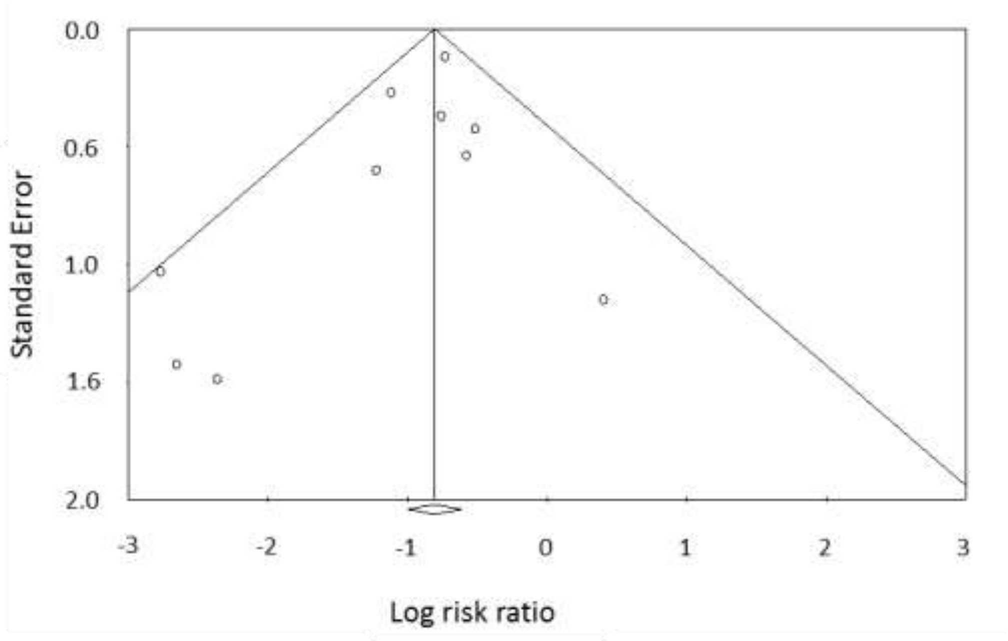

Figure S3. Funnel plot for meta-analysis of studies comparing universal screening vs risk-based protocols. Egger's regression test did not show statistically significant asymmetry of the funnel plot (2-tailed p = 0.180).
