# Supplementary material for: Universal screening versus risk‐based protocols for antibiotic prophylaxis during childbirth to prevent early‐onset group B streptococcal disease: a systematic review and meta‐analysis
Source: BJOG. 2020 Feb 4;127(6):680–91. doi: 10.1111/1471-0528.16085 (PMC7187465; doi:10.1111/1471-0528.16085)
Supplement: Supplementary file 4 — Table S1. Search protocol, MeSH = Medical Subject Headings [file BJO-127-680-s004.pdf]

Table S1. Search protocol, MeSH = Medical Subject Headings

	**Free terms**	**MESH terms**
Problem	Group B streptococcus; Streptococcus agalactiae	Streptococcus agalactiae [MeSH]
Patient group	Newborn; pregnancy	Pregnancy [MeSH]
Intervention protocol	Screening; Culture based policy; Risk based	
