# Supplementary material for: Universal screening versus risk‐based protocols for antibiotic prophylaxis during childbirth to prevent early‐onset group B streptococcal disease: a systematic review and meta‐analysis
Source: BJOG. 2020 Feb 4;127(6):680–91. doi: 10.1111/1471-0528.16085 (PMC7187465; doi:10.1111/1471-0528.16085)
Supplement: Supplementary file 5 — Table S2. Search strategy used to identify records in MEDLINE (final search March 2019). Similar queries were run in CINAHL and Embase [file BJO-127-680-s005.pdf]

Table S2. Search strategy used to identify records in MEDLINE (final search March 2019). Similar queries were run in CINAHL and EMBASE.

Search	Query	Items found
#5	Search ((((("streptococcus agalactiae") OR "group b streptococcus") OR streptococcus agalactiae[MeSH Terms])) AND ((("newborn") OR "pregnancy") OR "pregnancy"[MeSH Terms])) AND ((("screening") OR "culture based") OR "risk based") Filters: English & Dutch	665
#4	Search ((((("streptococcus agalactiae") OR "group b streptococcus") OR streptococcus agalactiae[MeSH Terms])) AND ((("newborn") OR "pregnancy") OR "pregnancy"[MeSH Terms])) AND ((("screening") OR "culture based") OR "risk based")	768
#3	Search (("screening") OR "culture based") OR "risk based"	555326
#2	Search (("newborn") OR "pregnancy") OR "pregnancy"[MeSH Terms]	1450974
#1	Search (("streptococcus agalactiae") OR "group b streptococcus") OR streptococcus agalactiae[MeSH Terms]	9702
