# Supplementary material for: Universal screening versus risk‐based protocols for antibiotic prophylaxis during childbirth to prevent early‐onset group B streptococcal disease: a systematic review and meta‐analysis
Source: BJOG. 2020 Feb 4;127(6):680–91. doi: 10.1111/1471-0528.16085 (PMC7187465; doi:10.1111/1471-0528.16085)
Supplement: Supplementary file 6 — Table S3. Articles excluded from analysis in the last step of the reviewing process [file BJO-127-680-s006.pdf]

Table S3. Articles excluded from analysis in the last step of the reviewing process

	Author	Year	Country	Reason for exclusion
1	Berardi et al.	2013	Italy	Problem with control group. Study only observes outcomes after adherence was improved
2	Carbonell-Estrany et al.	2012	Spain	Full text could not be obtained
3	Chang et al.	2017	Taiwan	Problem with control group and outcome. Onset of GBS infection not clearly defined (all infants <2 months included)
4	Chen et al.	2018	Australia	Problem with control group. Compares epochs that differ only in optimisation of established policy.
5	Ginsberg et al.	2014	Israel	Calculations through a model, lack of empirical data
6	Gopal Rao et al.	2017	UK	Same group has published a more extensive study with the same data
7	Homer et al.	2014	n/a	Review of guidelines, lack of empirical data
8	Kalliola et al	1999	Finland	Problem with control group. Only pre-policy period studies.
9	Khalil et al.	2018	Denmark	Outcome measure not EOGBS disease
10	Lin et al.	2011	Taiwan	Problem with co-intervention. Article discusses introduction of screening AND IAP in comparison of no screening AND no IAP.
11	Petersen et al.	2014	Denmark	Introduction of IAP coincides with introduction of new policy: co-intervention
12	Raychaudhuri et al.	2016	UK	Very small sample, eligibility criteria mostly unclear
13	Szymusik et al.	2013	Poland	Outcome measure not EOGBS disease
14	Van Dyke et al.	2009	USA	Problem with control group. Incidences in the control group (pre-screening) not clearly presented.
15	Wicker et al.	2018	Germany	Problem with the outcome. No possibility to distinguish between early and late onset disease
16	Daley et al.	2007	'Australasia'	Absence of sufficiently different intervention groups
