# Supplementary material for: Universal screening versus risk‐based protocols for antibiotic prophylaxis during childbirth to prevent early‐onset group B streptococcal disease: a systematic review and meta‐analysis
Source: BJOG. 2020 Feb 4;127(6):680–91. doi: 10.1111/1471-0528.16085 (PMC7187465; doi:10.1111/1471-0528.16085)
Supplement: Supplementary file 7 — Table S4. Risk of bias assessment according to ROBINS‐I, in studies comparing screening‐based protocols and risk‐based protocols (analysis 1). RoB, risk of bias; PB, performance bias [file BJO-127-680-s007.pdf]

Table S4. Risk of bias assessment according to ROBINS-I, in studies comparing screening-based protocols and risk-based protocols (analysis 1). RoB, risk of bias, PB, performance bias

Domain	Angstetra et al. 2007	Chen et al. 2004	Edwards et al. 2003	Gilson et al. 2000	Main&Slagle 2000	Schrag et al. 2002	Yücesoy et al. 2004	Eisenberg et al. 2005	Vergani et al. 2002	Ma et al. 2017	Gopal Rao et al. 2017
**Pre-intervention**											
Bias due to confounding	Moderate risk<br>Study identifies confounders demographics and preterm, but no other factors.	Serious risk<br>Study does not identify and deals with confounders sufficiently(demographics)	Serious risk<br>Study does not address confounders sufficiently (EOGBS is also secondary outcome)	Low-moderate risk<br>Groups are properly matched on demographics. Concurrent controls reduce RoB.	Moderate risk<br>Confounders are identified and dealt with. Although compliance seems a problem.	Moderate risk<br>Most confounders controlled for. Use of concurrent controls diminishes RoB.	Moderate risk<br>Some (e.g. preterm) confounders not controlled for.	Serious risk<br>Confounders exist and are not controlled for.	Moderate risk<br>Accurately dealt with most, but not preterm delivery.	Serious risk<br>Very little information is known about the risk-based group. Confounders suspected.	Low-Moderate risk<br>Confounders are dealt with and controlled for
Bias in selection of participants of the study	Low risk<br>Nearly all pregnant women in the population are included. NB: No prim. care clinics involved.	Moderate risk<br>Selection is expected to represent population (but show-up for screening is not researched)	Moderate risk<br>Selection is expected to represent population (although show-up not researched)	Serious risk<br>Hospital and satellite clinics are different intervention groups. Some explanation provided, but RoB remains.	Low risk<br>Authors present data on turn-up for screening (>96%), no indication of important bias.	Moderate risk<br>Selection bias: women in risk group had less prenatal care, and other ethnicity. Appropriately controlled for.	Critical risk<br>Selection bias in the screening group: are selected with threatening preterm delivery.	Moderate risk<br>Exclusions based on medical history pose a threat of bias. Weights are adjusted, but RoB remains	Moderate risk<br>Selection is expected to represent population; no information on women that showed for screening	Moderate risk<br>Representative group of women.	Low risk<br>Setting is the same in three periods.
**At intervention**											
Bias in classification of interventions	Moderate risk<br>Intervention (screen) described well, control intervention (risk) is not.	Moderate risk<br>Some risk of non-differential misclassification.	Serious risk<br>Interventions not defined accurately, and adherence to guidelines is not investigated.	Moderate risk<br>Retrospective design, but little risk of misclassification suspected	Low risk<br>Interventions are detailed. Implementing the protocols accurately and timely.	Moderate risk<br>There are problems in the retrospective classification of interventions. Efforts to control are done.	Low risk<br>Quasi-experimental, little bias from classification is suspected.	Serious risk<br>Some problems arise in retrospective classification of interventions.	Moderate risk<br>Interventions well defined. Problems during transition to new protocol suspected and not researched	Moderate risk<br>Risk-based policy is not defined well.	Moderate risk<br>Contamination could have happened in the transition period. Dealt with adequately.
**Post intervention**											
Bias due to deviations from intended interventions	No information<br>No information on adherence is provided. NB: care improves over time, not addressed (PB)	Serious risk<br>Suggestions of (non-usual practice) deviations exist; PB not addressed.	Serious risk<br>No information on compliance is provided, nor is PB (co-intervention) addressed.	Low risk<br>Concurrent controls. Compliance was retrospectively researched.	Low risk<br>Staff and patients were educated. Deviations as usual practice (intention-to-treat).	Low risk<br>Concurrent controls. Although contaminations are expected, dealt with.	Moderate risk<br>Concurrent controls reduced risk of deviation, but little information on adherence.	Moderate risk<br>Concurrent controls, yet little information provided on adherence. Cross-over is expected.	Moderate risk<br>Compliance mostly unclear. Could indicate a larger real effect.	Moderate risk<br>Information on rate of IAP in colonized women is presented. NB: care improves over time, not addressed	Low risk<br>Extensive information on real practice is presented. Cross-over design reduces performance bias.

Bias due to missing data	Moderate risk<br>Data were almost complete, althought reporting issues not addressed.	Moderate risk<br>Expected missing data (reporting problems) are not controlled for. RoB is limited.	Moderate risk<br>Expected missing data (reporting problems) are not controlled for. RoB is limited.	Moderate risk<br>Missing data are present, but RoB is not expected from this.	Low risk<br>Missing data are not expected comprehensive mother-child integral database.	Moderate risk<br>95% of selected births (in 5425 births representative sample) had abstracted charts.	Low risk<br>No indication that missing data would impose bias	No information	Moderate risk<br>Missing data addressed: not likely bias and would favour an increased effect.	No information<br>Missing data on risk-based period. Could not be solved by assessing earlier work.	Low risk<br>No indication that missing data would impose bias
Bias in measurements of outcomes	Moderate risk<br>Methods differ between groups, but are not likely to influence outcome	Low risk<br>Knowledge on intervention is unexpected. Cases are identified using lab data	Low risk<br>knowledge on intervention is unexpected. Cases are identified through lab records.	Low risk<br>Both culture-confirmed and clinical sepsis are included, still low risk of bias.	Low risk<br>Outcome measure was unlikely to be influenced by knowledge on intervention.	Low risk<br>Outcome measure was unlikely to be influenced by knowledge on intervention.	Serious risk<br>Cases are searched differently in groups. Outcome could be influenced.	Low risk<br>Outcome measure was unlikely to be influenced by knowledge on intervention.	Low risk<br>Outcome measure was unlikely to be influenced by knowledge on intervention.	Low risk<br>Outcome measure was unlikely to be influenced by knowledge on intervention.	Low risk<br>Outcome measure was unlikely to be influenced by knowledge on intervention.
Bias in selection of the reported results	Moderate risk<br>Outcomes correspond to standard incidence measures	Moderate risk<br>Outcomes correspond to standard incidence measures	Moderate risk<br>Outcomes correspond to standard incidence measures	Moderate risk<br>Outcomes correspond to standard incidence measures	Moderate risk<br>Multiple outcome measures are presented, alongside the standard measure for incidence.	Moderate risk<br>Overview of result is presented extensively, but in fractions instead of absolute numbers	Moderate risk<br>No indication that selection would have happened.	Serious risk<br>No indication that selection would have happened.	Serious risk<br>Comparison of maternal risk factors between different periods is missing.	Moderate risk<br>No indication that selection would have happened.	Moderate risk<br>No indication that selection would have happened.
**Overall risk of bias**	Moderate risk<br>The study has risks in the domain of confounding, although other domains are generally without problems	Serious risk<br>Study has critical problems in the domains of confounding and information on intervention status and missing data	Serious risk<br>Study has some problems due to possible confounders, and the absence of detailed methods.	Moderate risk<br>Generally sound study, but intervention groups differ. Problems are mostly dealt with.	Moderate risk<br>The study is sound for a non-randomized study. Problems are mostly dealt with.	Moderate risk<br>Study has some problems (mostly with assignment of intervention) but authors have adequately dealt with them to minimize the effect.	Critical risk<br>Too much risk of bias arises from selection of preterm delivering women into the screening group. Not controlled for.	Moderate risk<br>Study has concurrent controls (reducing time-dependent bias), but has some selection bias.	Moderate risk<br>Study has a good overall design but handling of confounders in: at least one important domain was not measured or controlled for.	Serious risk<br>Study has problems in defending validity of outcomes of the period with risk-based protocols. Methods and results missing.	Moderate risk<br>This is a sound study for an observational study. Adherence and demographics are very closely studied.
