# Supplementary material for: Universal screening versus risk‐based protocols for antibiotic prophylaxis during childbirth to prevent early‐onset group B streptococcal disease: a systematic review and meta‐analysis
Source: BJOG. 2020 Feb 4;127(6):680–91. doi: 10.1111/1471-0528.16085 (PMC7187465; doi:10.1111/1471-0528.16085)
Supplement: Supplementary file 8 — Table S5. Risk of bias assessment: studies assessing introduction of either of the two protocols compared with a period/area of ‘no policy’ (analysis 2) [file BJO-127-680-s008.pdf]

Table S5. Risk of bias assessment: studies assessing introduction of either of the two protocols compared to a period/area of 'no policy' (analysis 2)

Domain	Darlow et al. 2016	Håkansson et al. 2017	Bekker et al. 2014	O' Sullivan et al. 2019	Phares et al. 2008	Hung et al. 2018
**Pre-intervention**						
Bias due to confounding	Serious risk<br>Confounders are adressed but not sufficiently dealt with.	Serious risk<br>Not all confounders (i.e. demographic change) addressed. This might influence outcomes.	Moderate risk<br>Although confounders are present, (some) evidence of their limited influence on the effect is presented	Moderate risk<br>Some confounders are expected, most are addressed sufficiently. Some missing information from the previous studies.	Moderate risk<br>Confounders identified. Discussion provides suggestion that in both cases true effect would be larger if controlled for.	No information<br>Too little information on the former period of observation is provided; period 2 is well done.
Bias in selection of participants of the study	Low risk<br>Setting in hospitals and community care. Problems in reporting by clinicians is not likely to be related to intervention or outcome	Low risk<br>Nearly all women and births in Sweden are likely to be included in the study.	Moderate risk<br>Information is retrieved from a national surveillance, which covers most but not all cases. Assignment to intervention was clear.	Moderate risk<br>Some selection bias might exist, Could imply a reduced true effect.	Moderate risk<br>Cases might be better reported in some states than in other. Some states were included after guidelines. Statistically controlled for, but not in main outcomes	Serious risk<br>Problems exist: only screened women are included in period 2. NB: screening was made free of charge immediately.
**At intervention**						
Bias in classification of interventions	Moderate risk<br>Due to the prospective study, misclassification not expected. Yet, some hospitals employ other policy.	Low risk<br>It is clear which clinic switched to the nationally promulgated policy. Despite retrospective design, little bias expected.	Low risk<br>It is clear when institutions switched to the new protocol. Despite retrospective design, little bias expected.	Low risk<br>It is clear when institutions switched to the new protocol. Because time periods are far apart, little bias expected.	Low risk<br>Clearly demarcated periods, little bias in classification expected.	No information<br>Very little information is provided on earlier periods, so it is not possible to make an estimation.
**Post-intervention**						
Bias due to deviations from intended interventions	Serious risk<br>Little information, but bias due to improved health care can be expected.	Moderate risk<br>Implementation of guidelines gradual over the two periods. Some control for this is in place.	No information<br>Adherence is not studied, no data.	Moderate risk<br>Some suggestion that adherence to policy was better in second period. Real effect would be increased.	No information<br>Rates of IAP are unclear	No information

Bias due to missing data	Moderate risk<br>Little information is known on IAP administration overall and on compliance. Only confirmed (lab) GBS included in main outcome.	Low risk<br>Cases of clinical sepsis (non-confirmed) are researched in the study, but not the main outcomes. Outcomes are similar	Moderate risk<br>Estimation of infants' ages was suboptimal. Possible bias is addressed in discussion.	Moderate risk<br>Not all risk factors were recorded in period 1. Not expected to affect the outcome.	Moderate risk<br>Inherent underreporting is a risk of bias. Would only increase observed effect.	Serious risk<br>As only women who undergo screening are included in the study, underreporting is likely
Bias in measurements of outcomes	Serious risk<br>5 of 56 cases in period 1 were measured by methods no longer employed in period 2. This could increase the reported effect.	Moderate risk<br>While laboratory-confirmed GBS is not influenced by the outcome, the more subjective assessment of 'clinical sepsis' might. Authors address subgroups.	Moderate risk<br>Reporting of cased might have improved over the years, yet levels of E.Coli stayed constant making an effect of this bias less likely.	Low risk<br>Although various methods for the yielding of records for cases were employed, outcome is based on laboratory-confirmed cases.	Low risk<br>Knowledge of intervention is not expected to have a large effect on outcome.	Low risk<br>Measurement through lab data; knowledge of intervention is not expected to have a large effect on outcome.
Bias in selection of the reported results	Moderate<br>Outcomes correspond to standard incidence measures	Moderate<br>Not possible to assess, but little selection of results is suspected, as main outcome measures are present.	Moderate<br>Outcomes correspond to standard incidence measures	Low risk<br>Many results are presented comprehensively, in accordance to earlier study.	No information	No information
**Overall risk of bias**	Moderate-serious risk<br>In general sound, but controlling for confounders is not sufficient.	Moderate-Serious risk<br>In general sound, but controlling for confounders is not sufficient.	Moderate risk<br>Although inherent problems in this retrospective study exist, they are dealt with in the discussion section.	Moderate risk<br>Despite inherent flaws of the design, controlling measures are in place. Authors address effects of possible confounders in the discussion.	Moderate risk<br>Although populations between intervention groups seem to differ, authors explained possible consequences well. In general, many factors are investigated, reducing overlooked risk of bias.	No information<br>Although the analyses on the current incidence and current risk factors is sound, risk of bias in claims on improvement compared to earlier periods cannot be confirmed.
