# Supplementary material for: Universal screening versus risk‐based protocols for antibiotic prophylaxis during childbirth to prevent early‐onset group B streptococcal disease: a systematic review and meta‐analysis
Source: BJOG. 2020 Feb 4;127(6):680–91. doi: 10.1111/1471-0528.16085 (PMC7187465; doi:10.1111/1471-0528.16085)
Supplement: Supplementary file 9 — Table S6. Numbers needed to screen (NNS) as calculated from the data in the included studies [file BJO-127-680-s009.pdf]

Table S6. Numbers needed to screen (NNS) as calculated from data in the included studies. Interventions were either screening-based or risk-based protocols.

	Screening versus risk	Risk versus no policy	Screening versus no policy
Angstetra et al. 2007	1190		
Bekker et al. 2014		579*	
Chen et al. 2005	1429		
Darlow et al. 2016		3704	
Edwards et al . 2003	1471		
Eisenberg et al. 2005	2222		
Gilson et al. 2000	671		
Gopal Rao et al. 2017	1515		
Håkansson et al. 2017		10000	
Hung et al. 2018			1250
Ma et al. 2018			
Main & Slagle 2000	971		
O'Sullivan et al. 2019		11111*	
Phares et al. 2008			7692
Schrag et al. 2002	2857		
Vergani et al. 2002	3333		
Yücesoy et al. 2004	-200**		
**Pooled NNS**	1875	Not available	Not available

*'no policy' was superior to risk-based protocols **risk-based was superior to screening
