# Supplementary material for: Universal screening versus risk‐based protocols for antibiotic prophylaxis during childbirth to prevent early‐onset group B streptococcal disease: a systematic review and meta‐analysis
Source: BJOG. 2020 Feb 4;127(6):680–91. doi: 10.1111/1471-0528.16085 (PMC7187465; doi:10.1111/1471-0528.16085)
Supplement: Supplementary file 10 — Table S7. Secondary outcomes with weighted means [file BJO-127-680-s010.pdf]

Table S7. Secondary outcomes with weighted means. Estimated rate of vaginal colonisation in the population, the portion of parturients treated with antibiotic prophylaxis under different policies, and the portion of resistant pathogens isolated from clinically ill infants. AB, antibiotics; GBS, group B streptococcus.

Authors	1. Estimated prevalence of GBS colonization in pregnant women	2. Rate of AB use in screening era (in all pregnant women)	2.Rate of AB use in risk-based era (in all pregnant women)	3.GBS isolates resistant to penicillin/ampicillin	3.GBS isolates resistance to erythromycin	3.GBS isolates resistance to clindamycin
Angstetra et al. 2007	24%					
Chen et al. 2005				0%	14% (highest)	
Edwards et al . 2003				0%	0%	
Eisenberg et al. 2005	24%					
Gilson et al. 2000	13.3%					
Gopal Rao et al. 2017	29%					
Ma et al. 2018	21.8%*					
Main & Slagle 2000	13.4%	26%	25%**	0%		
Schrag et al. 2002	24%	31%**	29%**			
Vergani et al. 2002	18%	28%				
Yücesoy et al. 2004	7%	16%	21.2%			
Bekker et al 2014						
Darlow et al. 2016						
Håkansson et al. 2017						
Hung et al 2018	19.60%*					
O'Sullivan et al. 2019				0%	25%	17%
Phares et al. 2008				0%	32%	15%
**Weighted mean**	**23%**	**31%**	**29%**	**0%**	**19%**	**16%**

*Excluded from weighted mean due to lack of data **Computed rates based on prophylaxis indications and an assumption of near-perfect compliance.
