# Supplementary material for: Universal screening versus risk‐based protocols for antibiotic prophylaxis during childbirth to prevent early‐onset group B streptococcal disease: a systematic review and meta‐analysis
Source: BJOG. 2020 Feb 4;127(6):680–91. doi: 10.1111/1471-0528.16085 (PMC7187465; doi:10.1111/1471-0528.16085)
Supplement: Supplementary file 11 — Table S8. Missed cases in the included studies [file BJO-127-680-s011.pdf]

Table S8. Missed cases in the included studies. Three groups of cases are included based on policy and maternal indication for prophylaxis.

Authors	Risk-based period: of the GBS infants, how many were born to mothers without risk factors?	Screening period: of the GBS infants, how many were born to mothers with negative screening result?
Angstetra et al. 2007		
Chen et al. 2005		
Edwards et al . 2003		
Eisenberg et al. 2005	58%	40%
Gilson et al. 2000	75%	0%
Gopal Rao et al. 2017	11%	0%
Ma et al. 2018		41.3%
Main & Slagle 2000	46% *	
Schrag et al. 2002	62% **	
Vergani et al. 2002	75%	17%
Yücesoy et al. 2004	66%	
Bekker et al 2014		
Darlow et al. 2016	45%	
Håkansson et al. 2017	43%	
Hung et al 2018		58%
O'Sullivan et al. 2019	40% (lowest)	
Phares et al. 2008		
**Weighted mean**	41%	24%

GBS, Group B Streptococcus.

*only cases in term newborns are included ** both cases under screening and risk-based protocols taken together.
